# Red ginseng dietary fiber promotes probiotic properties of *Lactiplantibacillus plantarum* and alters bacterial metabolism

**DOI:** 10.3389/fmicb.2023.1139386

**Published:** 2023-03-06

**Authors:** Hyeon Ji Jeon, Seung-Hwan You, Eoun Ho Nam, Van-Long Truong, Ji-Hong Bang, Yeon-Ji Bae, Razanamanana H. G. Rarison, Sang-Kyu Kim, Woo-Sik Jeong, Young Hoon Jung, Minhye Shin

**Affiliations:** ^1^Food and Bio-Industry Research Institute, School of Food Science and Biotechnology, College of Agriculture and Life Sciences, Kyungpook National University, Daegu, Republic of Korea; ^2^Laboratory of Efficacy Research, Korea Ginseng Corporation, Daejeon, Republic of Korea; ^3^Department of Microbiology, College of Medicine, Inha University, Incheon, Republic of Korea; ^4^Department of Biomedical Sciences, Program in Biomedical Science and Engineering, Inha University, Incheon, Republic of Korea

**Keywords:** red ginseng, dietary fiber, probiotics, prebiotics, metabolomics

## Abstract

Korean red ginseng has been widely used as an herbal medicine. Red ginseng dietary fiber (RGDF) is a residue of the processed ginseng product but still contains bioactive constituents that can be applied as prebiotics. In this study, we evaluated changes on fermentation profiles and probiotic properties of strains that belong to family *Lactobacillaceae* with RGDF supplementation. Metabolomic analyses were performed to understand specific mechanisms on the metabolic alteration by RGDF and to discover novel bioactive compounds secreted by the RGDF-supplemented probiotic strain. RGDF supplementation promoted short-chain fatty acid (SCFA) production, carbon source utilization, and gut epithelial adhesion of *Lactiplantibacillus plantarum* and inhibited attachment of enteropathogens. Intracellular and extracellular metabolome analyses revealed that RGDF induced metabolic alteration, especially associated with central carbon metabolism, and produced RGDF-specific metabolites secreted by *L. plantarum*, respectively. Specifically, *L. plantarum* showed decreases in intracellular metabolites of oleic acid, nicotinic acid, uracil, and glyceric acid, while extracellular secretion of several metabolites including oleic acid, 2-hydroxybutanoic acid, hexanol, and butyl acetate increased. RGDF supplementation had distinct effects on *L. plantarum* metabolism compared with fructooligosaccharide supplementation. These findings present potential applications of RGDF as prebiotics and bioactive compounds produced by RGDF-supplemented *L. plantarum* as novel postbiotic metabolites for human disease prevention and treatment.

## Introduction

Ginseng is the root of plants in the genus *Panax* and has been widely used as an herbal medicine in Eastern Asia (So et al., 2018). It is typically characterized by the presence of ginsenosides, which are the main bioactive components with antioxidant, anti-proliferative, and neuroprotective properties (Tam et al., 2018). In recent years, the biological properties of ginseng have been extensively demonstrated; these include enhanced immune system performance and memory, and improved blood circulation (Geng et al., 2010; Cho et al., 2018; Su et al., 2022).

Korean red ginseng (*Panax ginseng* C.A. Meyer) is a processed product made by the repetitive steaming and drying of fresh ginseng to extend shelf life, reduce toxic effects, and enhance biological benefits (He et al., 2018). Red ginseng is traditionally consumed as a water extract containing a high concentration of ginsenosides. The residues are usually discarded, but they still contain bioactive constituents, such as unextracted ginsenosides, acidic polysaccharides, mineral elements, and dietary fiber (Yu et al., 2020). Many attempts to make the most use of these residues have included pharmaceutical, health functional foods, and cosmetics applications (Truong and Jeong, 2022).

Dietary fibers are carbohydrate polymers from plant-derived foods that are not digested by human enzymes or absorbed in the gut. Polymers contribute to human gut health by increasing stool weight and regularity, thickening the contents of the intestinal tract, and promoting growth of gut microbes (Makki et al., 2018). In particular, dietary fiber can be a good fermentable source for bacteria within the large intestine and influences the composition of bacterial communities as well as microbial metabolic activities producing fermentative end products, such as short-chain fatty acids (SCFAs). These prebiotic fermentable fibers promote metabolic interactions among bacterial communities that cross-feed probiotics and inhibit the proliferation of pathogens (Holscher, 2017).

*Lactobacillaceae* (including newly defined *Lactobacillus*-associated genera by taxonomic changes such as *Lactiplantibacillus* and *Limosilactobacillus*) and *Bifidobacteria* are the most well-known genera of probiotic organisms that normally reside in human gastrointestinal tracts. Probiotics are live microorganisms which benefit the host by producing useful physiologically bioactive compounds. These compounds have immunomodulatory, anti-carcinogenic, anti-aging, and antimicrobial effects in hosts. However, the use of these compounds is currently limited by a lack of knowledge of their molecular mechanisms, strain specific behaviors, and safety (Bourebaba et al., 2022). To address these limitations, recent studies have focused on elucidating microbial metabolism and discovering postbiotic molecules, which are defined as metabolic products secreted by probiotics in cell-free supernatants (Nataraj et al., 2020).

Metabolomics is the systematic study of unique chemical molecules, termed metabolites, generated by specific cellular processes (Jordan et al., 2009). Metabolomic data are used for phenotyping molecular interactions, identifying potential biomarkers, and discovering new therapeutic targets. In this study, we aimed to find an effective strategy for utilizing processed red ginseng residue as a prebiotic dietary fiber source and evaluated its prebiotic properties on the changes in growth, metabolism, and epithelial attachment ability of probiotic *Lactobacillaceae* strains. Comprehensive metabolomic analyses were performed to investigate the effects of red ginseng dietary fiber (RGDF) on bacterial metabolism and to discover novel bioactive compounds secreted by the RGDF-supplemented probiotic strain.

## Materials and methods

### Bacterial strains and media

*Limosilactobacillus reuteri* KCTC 3594 and *Lactiplantibacillus plantarum* KCTC 3108 were obtained from the Korean Collection for Type Cultures (KCTC, Jeongeup, Republic of Korea). The strains were pre-cultured in 50 ml of MRS broth (BD Difco, Franklin Lakes, NJ, USA) in 50 ml conical tubes and were incubated at 37°C without shaking (Biofree, Seoul, Republic of Korea) overnight. Cultures of the probiotic strains were then generated at 37°C in 50 ml of MRS broth supplemented with 0.5, 1, or 2% RGDF (Korea Ginseng Corporation, Daejeon, Republic of Korea). Composition of MRS broth is as follows: 10 g/L proteose peptone, 10 g/L beef extract, 5 g/L yeast extract, 20 g/L dextrose, 1 g/L polysorbate 80, 2 g/L ammonium citrate, 5 g/L sodium acetate, 0.1 g/L magnesium sulfate, 0.05 g/L manganese sulfate, and 2 g/L dipotassium phosphate.

### Preparation of RGDF

The residue remaining after water extraction of red ginseng at 87°C for 24 h was provided by Korea Ginseng Corporation (Daejeon, Republic of Korea). RGDF was prepared from the residue by drying it at 115°C and pulverizing it to 50 mesh. The physicochemical characteristics of RGDF were analyzed as previously reported (Yu et al., 2022), and same RGDF material was used in this study.

### Measurement of bacterial growth and cell mass

Colony forming units per ml of probiotic strains cultured in MRS broth or in MRS supplemented with 0.5, 1, or 2% of RGDF were measured by serial dilution at 0, 3, 6, 12, and 24 h. Dry cell weight of strains at 24 h was measured by collecting cell pellets by centrifugation at 4,000 rpm and 4°C for 15 min, washing the pellets three times with 10 ml of 1% (w/v) phosphate buffered saline, and drying in a dry oven (JS Research Inc., Natural Convection Oven, Gongju, Republic of Korea) at 70°C for 24 h. pH of the cultured media was measured using a pH meter (Ohaus, Parsippany, NJ, USA).

### Analysis of SCFAs

The concentrations of formic, acetic, propionic, and butyric acids were measured by high-performance liquid chromatography (HPLC) using the LC-6000 system (FUTECS, Daejeon, Republic of Korea). Each 1.5 ml of culture medium was collected by centrifugation (Eppendorf, Hamburg, Germany) at 13,000 rpm for 5 min at 4°C and filtered through a 0.45 μm nylon membrane filter. HPLC analysis was performed using an Aminex HPX-87X organic acid column (Bio-Rad, Hercules, CA, USA) with 0.005 M H_2_SO_4_ as the mobile phase, with a constant elution flow of 0.5 ml/min at 55°C.

### Carbon source utilization analysis

An API kit (BioMérieux, Marcy l’Étoile, France) was used to compare the ability of probiotic strains to utilize the particular carbon source. Inoculation samples were prepared by collecting cultured strains from each medium that had a turbidity greater than a McFarland standard of 4. One hundred microliters of sample were inoculated into the API strip and incubated at 37°C for 4 h. After incubation, the reagents were added for reading, incubated for 10 min, and exposed to strong light at 1,000 W for 10 s to decolorize any excess reagent. Identification and interpretation were performed using the numerical profiles.

### Analysis of bacterial attachment to intestinal epithelial cells

Caco-2 cell line was procured from the American Type Culture Collection (ATCC, Manassas, VA, USA) and cultured in Minimum Essential Medium (MEM) supplemented with 10% fetal bovine serum, 100 U/ml penicillin, and 100 μg/ml streptomycin at 37°C in a 5% CO_2_ atmosphere. *Escherichia coli*, purchased from ATCC, were grown in Luria Broth (LB) overnight. *E. coli* and RGDF-pretreated probiotic strains were harvested by centrifugation at 5,000 rpm for 10 min, washed twice with sterile PBS, and re-suspended in serum and antibiotic-free MEM.

For adhesion assay, Caco-2 monolayer was inoculated with approximately 10^8^ CFU/ml of *L. reuteri* or *L. plantarum* and incubated for 2 h in a 5% CO_2_ incubator. After incubation, the monolayers were washed three times with sterile PBS to remove non-adherent bacteria. The Caco-2 cells with adherent bacteria were detached using trypsin-EDTA solution. Bacterial counts were performed by the colony counting method on MRS agar plates. Adhesion result was expressed as the percentage of the bacteria adhered divided by the initial count of bacteria added.

For competition assay, approximately 10^8^ CFU/ml of each probiotic strain and *E. coli* was co-incubated with Caco-2 monolayer for 1 h in a 5% CO_2_ incubator. Non-bounded bacteria were then washed three times with sterile PBS and the Caco-2 cells with adherent bacteria were detached using trypsin-EDTA solution. The number of viable adhering *E. coli* was determined using the colony counting method on LB agar plates. The competition index was expressed as the percentage inhibition of *E. coli* adhesion in the presence of each probiotic strain divided by the adhesion of bacteria in the absence of probiotic strains.

### Metabolome analysis

GC-MS has advantages of a greater chromatographic resolution compared to LC-MS and large spectral libraries, although the chemical range of metabolome coverage is narrower than LC-MS (Aretz and Meierhofer, 2016). Recently, more researches have used LC-MS to detect more peaks, but most of the identified metabolites by LC-MS are considerably overlapped with GC-MS except for lipid molecules having large molecular weights. GC-MS has been the most commonly used technique for metabolite profiling because of its hard ionization method which is highly reproducible and easy for metabolite annotation, and it still has been widely applied for metabolite profiling and identification (Baiges-Gaya et al., 2023; Kurbatov et al., 2023; Neag et al., 2023).

For metabolome analysis, each intracellular and extracellular metabolites were measured in *L. plantarum* and *L. reuteri* grown in MRS medium with different supplementation of RGDF, fructooligosaccharides, or without addition. To extract intracellular and extracellular metabolites from the probiotic strains, each strain was cultured in 15 ml of medium until the mid-exponential phase determined by measuring its growth curve. Fifteen milliliters of each probiotic culture was centrifuged at 4,000 rpm for 15 min at 4°C. The supernatant was filtered through a 0.2 μm syringe filter composed of polyvinylidene fluoride for the extraction of extracellular metabolites. Aliquots (750 μL) of the filtered supernatants was mixed with 2.25 ml of 4°C methanol (GC-grade 100%; Sigma-Aldrich, St. Louis, MO, USA) and vortexed for 1 min. The mixtures were centrifuged at 13,000 rpm and 4°C for 10 min, and 0.1 ml of each supernatant was collected and completely dried using a Spin Driver Lite VC-36R (TAITEC Corporation, Koshigaya City, Saitama, Japan) at 2,000 rpm for 24 h.

To extract intracellular metabolites from the cell pellet, 1 ml of 0.9% cold NaCl (w/v) was added to the pellet and filtered through a 0.2 μm syringe filter. Then, it was transferred to a 15 ml conical tube and washed twice with 10 ml of 0.9% cold NaCl (w/v). The final washed pellet was mixed with 2 ml methanol, vortexed for 10 min, and sonicated for 1 min on ice. The material was mixed with 2 ml of chloroform, vortexed for 10 min, and sonicated for 1 min with ice. Water (1.8 ml) was added and vortexing and sonication were repeated. The final mixtures were centrifuged at 13,000 rpm and 4°C for 10 min, and 0.1 ml the upper supernatant layer of each was collected and completely dried using the aforementioned Spin Driver Lite VC-36R under same conditions to extract extracellular metabolites. Methoxymation and silylation were performed for the derivatization of intracellular and extracellular metabolites. For methoxymation, 10 μL containing 20,000 ppm methyl hydroxyl chloride amine in pyridine was mixed with each dried sample and incubated at 30°C for 90 min. Next, 45 μL of N-methyl-N-trimethylsilyl-trifluoroacetamide (Fluka, Buchs, Switzerland) and 30 μL of fluoranthene as internal standard were added, vortexed for silylation, and incubated at 37°C for 30 min. The derivatized sample was transferred to a gas chromatography (GC) vial with an insert.

Gas chromatography was performed using a Crystal 9000 chromatograph (Chromatotec, Val-de-Virvée, France) coupled with a Chromatotec-crystal mass spectrometer (photomultiplier detector) for the analysis of untargeted metabolites. One microliter of the derivatized sample was injected into a VF-5MS GC column (Agilent, Santa Clara, CA, USA). The oven temperature was initially 50°C for 2 min, then increased to 320°C at a rate of 5°C/min, and held at 320°C for 10 min. The helium carrier gas flowed at a rate of 1.5 ml/min.

### Statistical analysis

For the deconvolution of the mass spectrometry (MS) data and identification of metabolites, MS-DIAL ver. 4.70 was used. All records of the Fiehn RI Library were used to identify metabolites by matching the MS peaks. Based on n-alkane mixture, the calculation of retention index was conducted using Kovats retention index formula:


$$\text{RI}=100\times \left(\text{n}\right)+100\times \left(m-n\right)\times \frac{t\,r\,i-t\,r\,n}{t\,r\,m-t\,r\,n}$$

where RI, retention index of a metabolite “i”; n, carbon number of the alkane which elutes before “i”; m, number of carbons of the alkane which elutes after “i”; tri, retention time of “i”; trn, retention time of the alkane which elutes before “i”; and trm, retention time of the alkane which elutes after “i”. Retention index of each metabolite was compared with the value of standards registered in NIST 2020 Mass Spectral Library (NIST, Gaithersburg, MD, USA), and metabolites were identified based on the retention indices and mass fragmentation profiles.

Uni- and multi-variance analyses, principal component analysis (PCA), hierarchical clustering analysis, and metabolite set enrichment analysis (MSEA) were performed using MetaboAnalyst (Ver. 5.0). Network analysis, such as MetaMapp, was performed using Cytoscape software.

## Results

### RGDF supplementation promotes SCFA production and carbon source utilization in *L. plantarum*

Red ginseng contains ginsenosides that have important pharmacological roles in cancer, diabetes, and aging (Yuan et al., 2012; Yu et al., 2020; Hong et al., 2022). The by-products of the processing red ginseng still contain several types of bioactive components, such as acidic polysaccharides and dietary fiber, as well as the remaining ginsenosides (Park and Kim, 2006). RGDF is a byproduct composed of approximately 31% dietary fiber (314.3 mg/g) and 0.66% ginsenoside (6.63 mg/g of total ginsenosides) (Yu et al., 2022).

**FIGURE 1:**
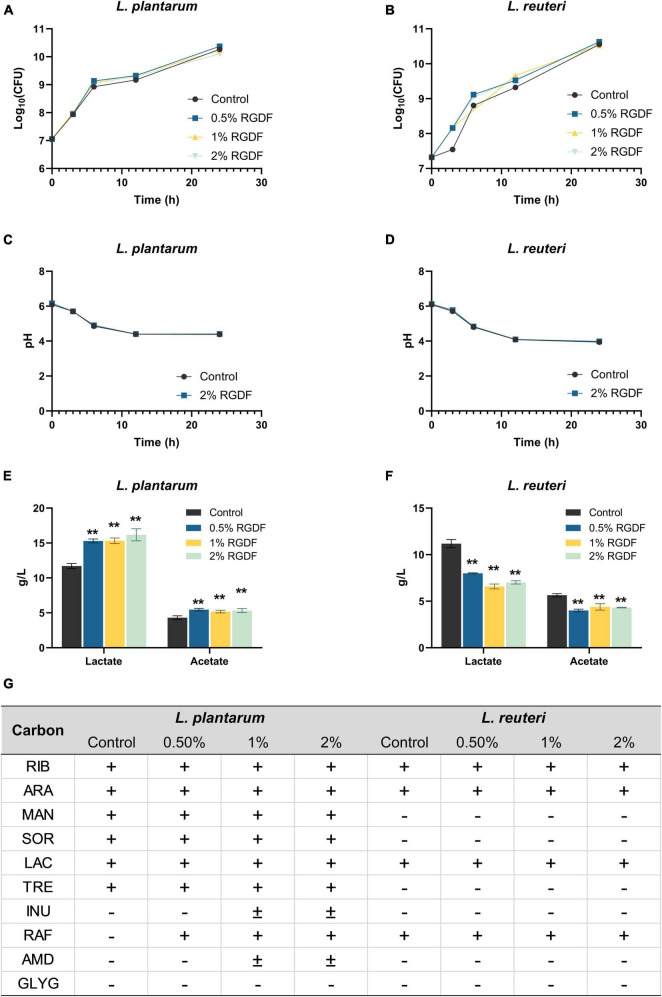
Fermentation profiles of *L. plantarum* and *L. reuteri*. **(A,B)** Bacterial growth, **(C,D)** pH of cultured media, **(E,F)** lactate and acetate production, and **(G)** carbon source utilization. Differences were indicated at a significance level of 95% (*) and 99% (**), as determined by one-way ANOVA with Dunnett’s *post-hoc* analysis. Error bars represent standard deviation (SD). RGDF, red ginseng dietary fiber; RIB, D-ribose; ARA, L-arabinose; MAN, D-mannitol; SOR, D-sorbitol; LAC, D-lactose; TRE, D-trehalose; INU, inulin; RAF, D-raffinose; AMD, starch; GLYG, glycogen; +, positive; –, negative.

Since dietary fibers are well-known prebiotic ingredients for bacterial growth promotion and probiotic functionality, we first screened the effects of RGDF on metabolic profiles of probiotic strains, including *L. reuteri*, *L. plantarum*, *Lactobacillus acidophilus*, *Lacticaseibacillus casei*, and *Lactococcus lactis* (Supplementary Table 1). We selected two probiotic strains, *L. plantarum* and *L. reuteri*, which were most positively and negatively affected, respectively, by RGDF supplementation. Although RGDF supplementation slightly enhanced the growth of both probiotic *Lactobacillaceae* strains, the difference was not significant compared with control (Figures 1A, B). The pH change of cultured media also was not different between control and RGDF supplementation. To reveal possible associations between RGDF and probiotic functionality, we next measured the production of SCFAs and carbon source utilization profiles with RGDF. *L. plantarum* enhanced the production of SCFAs, specifically lactate and acetate, with RGDF supplementation in a dose-dependent manner. *L. reuteri* reduced the production of these metabolites (Figures 1C, D). RGDF also improved the carbon source utilization ability of *L. plantarum* but had no effect on *L. reuteri* (Figure 1E). Thus, RGDF supplementation can promote the production of beneficial metabolites (lactate and acetate) and carbon source utilization by *L. plantarum*.

### RGDF supplementation promotes gut epithelial adhesion of *L. plantarum* and protects against enteropathogens

**FIGURE 2:**
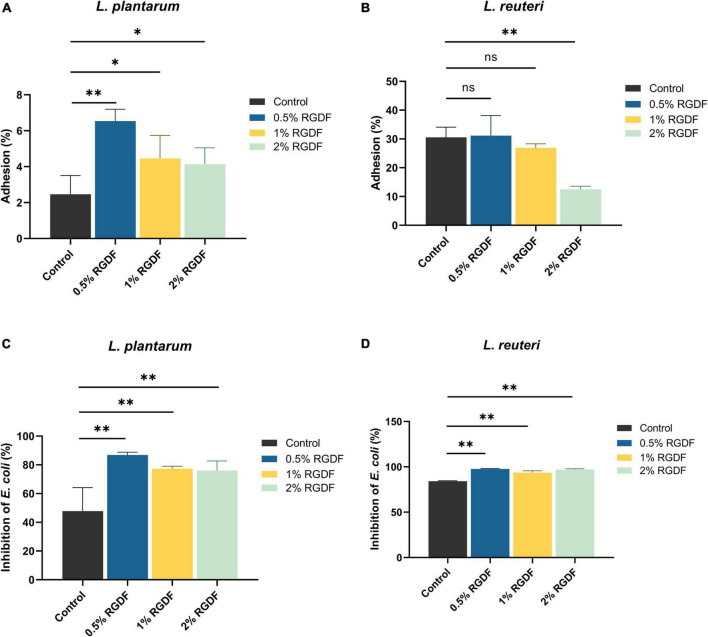
Gut epithelial adhesion **(A,B)** and inhibition of *E. coli* attachment **(C,D)** of *L. plantarum* and *L. reuteri*. Differences were indicated at a significance level of 95% (*) and 99% (**), as determined by one-way ANOVA with Dunnett’s *post-hoc* analysis.

Dietary fibers help maintain intestinal homeostasis by promoting probiotics, limiting the growth and adhesion of pathogenic microbes, and stimulating fiber-derived SCFA production (Cai et al., 2020). RGDF supplementation significantly increased the adhesion of *L. plantarum* to gut epithelial cells compared to the control. The adhesion was most pronounced in the presence of 0.5% RGDF (Figure 2A). Adhesion of *L. plantarum* and *L. reuteri* to the gut epithelium was decreased by adding RGDF (Figure 2B). *L. reuteri* is a probiotic that has a well-documented adhesive ability (approximately 30% in the control) (Gao et al., 2016). This behavior was confirmed in the present study; a high percentage of adhesion in the control was evident compared with *L. plantarum* (approximately 2% in the control).

To evaluate the competitive inhibitory effects of RGDF-supplemented strains on binding of enteropathogenic bacteria to the host epithelium, *E. coli* and RGDF-pretreated probiotic strains were co-incubated with Caco-2 monolayer (Figures 2C, D). Supplementation with RGDF increasingly reduced the *E. coli* attachment in the presence of both *L. plantarum* and *L. reuteri*; greater differences were observed in *L. plantarum*. Similar to the epithelial adhesion of *L. reuteri*, the strain showed a higher basal level of competitiveness against pathogen attachment than *L. plantarum*. However, addition of RGDF significantly improved adhesion of the gut epithelium and protected against *E. coli* attachment of *L. plantarum*, which can broaden the applicability of the strain as a probiotic. It is noted that several factors would affect epithelial adhesion of the strains including presence of surface proteins, auto-aggregation and bacterial surface hydrophobicity. Bacterial adhesion is based on non-specific physical interactions and aggregation abilities that also form a barrier preventing colonization of pathogens (Kos et al., 2003). Dell’Anno et al. (2021), showed that both *L. plantarum* and *L. reuteri* showed auto-aggregation and epithelial adhesion. *L. plantarum* and *L. reuteri* had higher hydrophobicity and greater auto-aggregation, respectively, reflecting their different colonizing ability. The collective findings indicate that RGDF supplementation promoted gut epithelial adhesion and had a protective role against enteropathogens in the presence of *L. plantarum*.

### RGDF supplementation alters intracellular metabolic profiles of *L. plantarum*, but not *L. reuteri*

**FIGURE 3:**
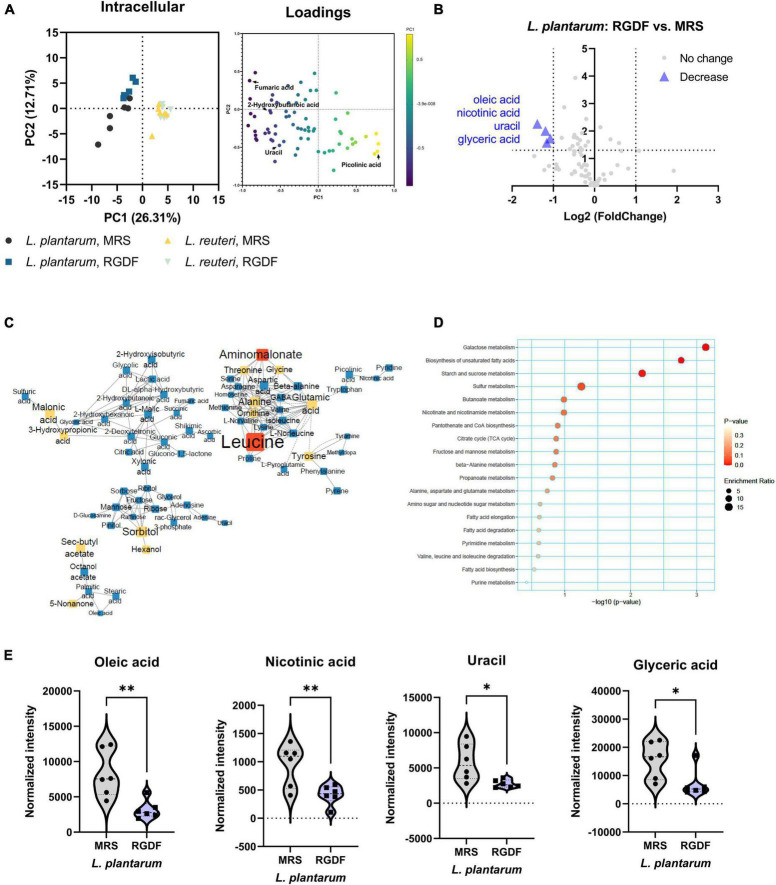
Intracellular metabolomic analysis of *L. plantarum* and *L. reuteri* cultured with 0.5% (w/v) RGDF compared to the control MRS broth. **(A)** Principle component analysis (PCA) score and loading plots. **(B)** Volcano plot of *L. plantarum*. Significantly decreased metabolites are indicated by blue triangles. **(C)** MetaMapp of *L. plantarum* culture with RGDF compared to the control MRS broth. Each node is a structurally identified metabolite. Blue nodes are decreased metabolites, and yellow nodes are unchanged metabolites. The size of nodes and labels reflect fold-changes and *p*-values by *t*-test, respectively. **(D)** MSEA of *L. plantarum*. **(E)** Normalized abundance of intracellular metabolites of *L. plantarum* cultured with 0.5% (w/v) RGDF compared to the control MRS broth. Data are expressed as violin plots of six determinations. Differences between metabolite abundances were all significant at a significance level of 95% (*) and 99% (**), as determined by the Student’s *t*-test.

Although both *L. plantarum* and *L. reuteri* utilize dietary fibers as prebiotics, our results indicate that RGDF supplementation was effective in *L. plantarum*, but not in *L. reuteri*. To identify the effects of RGDF on bacterial metabolism, we first determined the intracellular metabolome changes between RGDF supplementation and control in *L. plantarum* and *L. reuteri*. Total 106 of metabolites were identified including sugars, amino acids, fatty acids, organic acids, and polyamines (Supplementary Table 2). PCA results clearly showed metabolic alterations with 0.5% (w/v) RGDF supplementation in *L. plantarum*, while the metabolic profile of *L. reuteri* with RGDF was not different (Figure 3A). Loading of PC1 and PC2 indicated that fumaric acid (−0.834 at PC1), uracil (−0.924 at PC1), picolinic acid (0.791 at PC1), and 2-hydroxybutanoic acid (0.763 at PC1) were important metabolites determining the metabolic differences between *L. plantarum* and *L. reuteri*.

MetaMapp, a network graph of metabolites based on biochemical pathways and chemical and mass spectral similarities, displayed significantly altered metabolites (*p* < 0.05) with RGDF compared to the control in *L. plantarum* (Figure 3C). MSEA also supported the results of significantly altered bacterial metabolism, especially sugar (galactose, starch, and sucrose) metabolism and unsaturated fatty acid biosynthesis (Figure 3D). Considering the significant increase in lactate and acetate production and carbohydrate utilization in *L. plantarum* with RGDF (Figure 1), we suggest that glycolytic metabolic flow and membrane flexibility, respectively, can be affected by RGDF supplementation.

In addition, we compared the effect of RGDF on the intensity of each metabolite with that of the control using a volcano plot (Figure 3B). The intensities of the four metabolites (oleic acid, nicotinic acid, uracil, and glyceric acid) decreased after RGDF supplementation in *L. plantarum* (Figure 3E). The relative abundance of these metabolites was also significantly reduced by RGDF, verifying that metabolic processes associated with the four metabolites were specifically altered by RGDF (Supplementary Figure 1). Together, these findings suggest that *L. plantarum*, but not *L. reuteri*, is specifically affected by RGDF supplementation via central carbon metabolism.

### RGDF supplementation promotes biosynthesis of specific metabolites in *L. plantarum*

**FIGURE 4:**
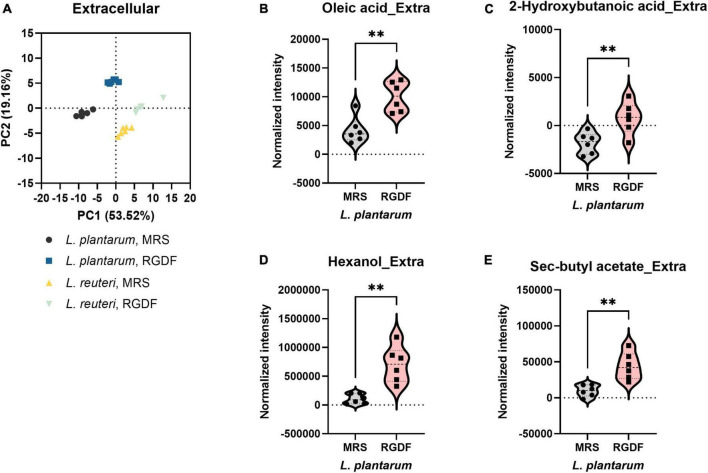
Extracellular metabolomic analysis of *L. plantarum* and *L. reuteri* cultured with 0.5% (w/v) RGDF compared to the control MRS broth. **(A)** Principle component analysis (PCA) score and loading plots. Normalized abundance of intracellular metabolites of *L. plantarum* **(B–E)** cultured with 0.5% (w/v) RGDF compared to the control MRS broth. Data are expressed as violin plots of six determinations. Differences between metabolite abundances were all significant at a significance level of 95% (*) and 99% (**), as determined by the Student’s *t*-test.

Postbiotics are nonviable bacterial metabolic products with biological activity in the host (Nataraj et al., 2020). These molecules have several advantages over probiotics with respect to safety and effectiveness, such as triggering only targeted responses by a defined mechanism, better accessibility of microbe-associated molecular patterns, and ease of production and storage (Nataraj et al., 2020). To systemically characterize postbiotic metabolites specifically produced by RGDF supplementation in *L. plantarum*, we further analyzed the extracellular metabolome in *L. plantarum* and *L. reuteri* grown with 0.5% RGDF, defined as the relative metabolite intensity in spent medium from bacterial culture to metabolite intensity in baseline medium (Jain et al., 2012). As shown in the PCA results, exometabolome profiles were clearly separated between the bacterial strains, as well as between the RGDF supplement and control (Figure 4A).

Red ginseng dietary fiber-specific bacteria-derived metabolites were distinguished from the media components based on three criteria: (1) the averaged value of metabolite intensity in the spent medium subtracted from its intensity in the uncultured medium should be positive; (2) the statistical significance between RGDF and control should be under the level of 95% confidence; and (3) the absolute change in metabolite intensity with RGDF compared to the control should be >2. Based on these criteria, we identified four *L. plantarum* metabolites (oleic acid, 2-hydroxybutanoic acid, hexanol, and sec-butyl acetate) biosynthesized specifically in response to the RGDF supplement (Figures 4B–E). The collective findings indicate that RGDF supplementation promoted the biosynthesis of specific metabolites in *L. plantarum*. These metabolites included oleic acid, 2-hydroxybutanoic acid, hexanol, and butyl acetate.

### RGDF supplementation has distinct effects on *L. plantarum* metabolism compared with fructooligosaccharide supplementation

Dietary fiber, a plant-derived component that cannot be completely digested by human enzymes, consists of non-starch polysaccharides, including cellulose and oligosaccharides (Veronese et al., 2018). Fructooligosaccharides (FOS) are dietary fibers composed of linear chains of fructose units linked by β-(2,1) bonds (Sabater-Molina et al., 2009). They naturally occur in plants, such as onion, chicory, and banana, and are increasingly used in food products because of their prebiotic effect, which stimulates the growth of probiotic gut microbiota (Sabater-Molina et al., 2009). To compare the effects of different type of dietary fibers on metabolic alteration in *L. plantarum*, we cultured *L. plantarum* on control MRS, MRS with 0.5% RGDF, and MRS with 0.5% FOS. Similar to the growth results of RGDF shown in Figure 1, supplementation with either RGDF or FOS did not have an effect on bacterial growth (Supplementary Table 3).

**FIGURE 5:**
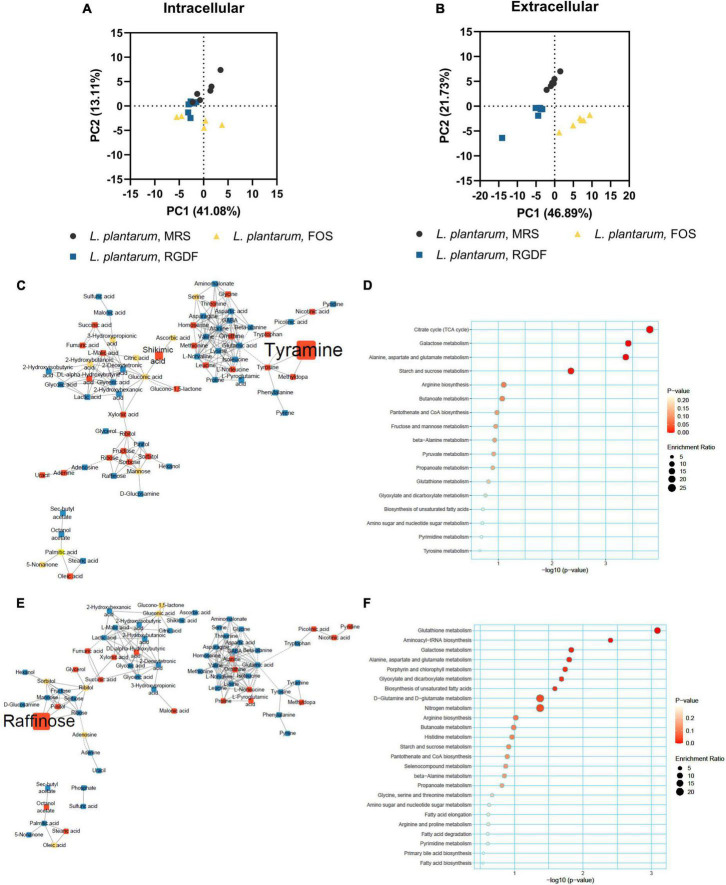
Metabolomic analysis of *L. plantarum* cultured with 0.5% (w/v) RGDF or 0.5% (w/v) FOS. Principle component analysis (PCA) score and loading plots of intracellular **(A)** and extracellular **(B)** metabolome. MetaMapp of *L. plantarum* cultured with FOS compared to the control MRS broth **(C)** and cultured with RGDF compared to FOS **(E)**. Each node is a structurally identified metabolite. Blue nodes are decreased metabolites, red nodes are increased metabolites, and yellow nodes are unchanged metabolites. The size of nodes and labels reflect fold-changes and *p*-values by *t*-test, respectively. MSEA of *L. plantarum* cultured with FOS compared to the control MRS broth **(D)** and cultured with RGDF compared to FOS **(F)**.

In contrast to the lack of observable differences in bacterial growth, the metabolome profile of *L. plantarum* supplemented with RGDF showed a transition between MRS and FOS in both intracellular and extracellular states (Figures 5A, B). Similar to the effect of RGDF shown in Figures 3C, D, FOS also decreased the abundance of specific metabolites in sugar and central carbon metabolism, while the abundance of leucine specifically increased with FOS supplementation compared to the control (Figure 5C). MSEA analysis indicated that the citrate cycle and its associated pathways, such as alanine, aspartate, and glutamate metabolism, as well as sugar metabolism, were altered by FOS treatment (Figure 5D). Comparison of the intracellular metabolite abundance of RGDF with FOS revealed that RGDF supplementation resulted in a decreased abundance of palmitic acid and stearic acid, while uracil, raffinose, ascorbic acid, and 2-hydroxybutanoic acid comparatively increased in RGDF (Figures 5E, F).

**FIGURE 6:**
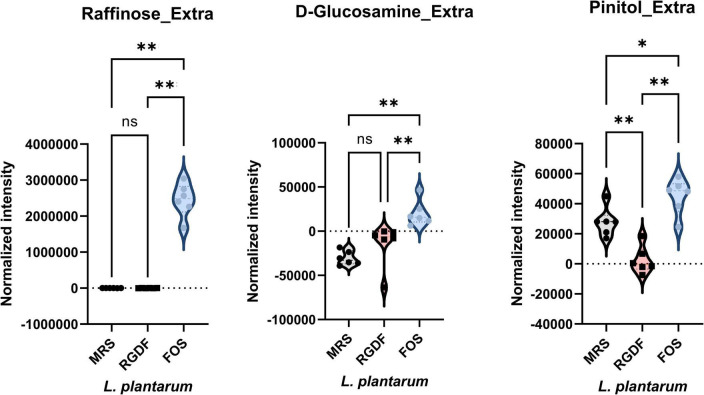
Normalized abundance of extracellular metabolites of *L. plantarum* cultured with 0.5% (w/v) RGDF or 0.5% (w/v) FOS. Data are expressed as violin plots of six determinations. Differences were indicated at a significance level of 95% (*) and 99% (**), as determined by one-way ANOVA with Tukey’s *post-hoc* analysis.

Next, we compared the extracellular metabolites differentially produced by FOS treatment to the control, applying the same criteria used for RGDF treatment (Figure 6). As expected, the culture supernatant of cells grown with FOS contained a significantly higher abundance of sugars and sugar derivatives than those grown with RGDF, including raffinose, D-glucosamine, and pinitol. Production of RGDF-specific metabolites, including oleic acid, 2-hydroxybutanoic acid, hexanol, and sec-butyl acetate, was not significantly induced by FOS, suggesting that the metabolism of these molecules is RGDF-specific. Thus, RGDF supplementation had distinct effects on *L. plantarum* metabolism compared with FOS supplementation.

## Discussion

Dietary fibers and the associated phytochemicals in ginseng-derived products provide various functional and health benefits. In this study, we evaluated the effects of RGDF as a prebiotic constituent on the physiological and metabolic alterations of probiotics. With RGDF supplementation in the growth media, *L. plantarum* showed the highest production of SCFAs, specifically lactate and acetate, and the most increased carbohydrate-fermenting capability compared with other probiotic *Lactobacillaceae* species, especially *L. reuteri*. In addition, RGDF improved gut epithelial adhesion of *L. plantarum* and protected against enteropathogens. Analysis of the intracellular metabolome of *L. plantarum* indicated decreases in metabolites of sugars and unsaturated fatty acids, and significant decreases in the abundance of oleic acid, nicotinic acid, uracil, and glyceric acid. RGDF supplementation also promoted the secretion of specific metabolites, such as oleic acid, 2-hydroxybutanoic acid, hexanol, and butyl acetate, in *L. plantarum*. Comparison of the metabolic alteration by red ginseng-derived dietary fiber with a representative dietary fiber, FOS, showed distinguishable effects between the two different types of fibers in *L. plantarum*.

Although dietary fibers generally promote probiotic growth, their effects are strain specific. Our results consistently revealed that RGDF supplementation improved the probiotic properties of *L. plantarum*, but not of *L. reuteri*. *L. plantarum*, unlike most probiotic *Lactobacillaceae* species, exhibits ecological and metabolic flexibility and thus maintains a diverse functional genome that facilitates the flexibility to colonize a variety of environments (Fidanza et al., 2021). For example, *L. plantarum* strains exhibit acid tolerance by inducing alterations in the fatty acid composition of the bacterial membrane upon exposure to low-pH conditions (Huang et al., 2016). Genome analysis of 165 *L. plantarum* strains revealed the presence of a large number of carbohydrates metabolizing genes and two-component systems and signal transduction systems regulating physiological processes, facilitating the adaptability of the species in various environments compared to other lactic acid bacteria and even among probiotic *Lactobacillaceae* strains (Cui et al., 2021). In addition, *L. plantarum* produces bacteriocins termed plantaricins, which can effectively inhibit enteropathogenic bacteria, such as *E. coli*, under specific circumstances (Pal and Srivastava, 2014). These findings based on the diverse functional genetic characteristics support our results that *L. plantarum* greatly modulates and improves their metabolic functions, including acid production, carbohydrate utilization, and inhibition of pathogen growth in the presence of RGDF.

To explain how RGDF promotes bacterial metabolic alterations in *L. plantarum*, but not in *L. reuteri*, and how the effects of RGDF are different from those of other dietary fibers, beyond the genetic flexibility of *L. plantarum*, we interpreted intracellular metabolic changes of *L. plantarum* and *L. reuteri* when supplied with RGDF and FOS. RGDF supplementation resulted in a significant decrease in the abundance of oleic acid, nicotinic acid, uracil, and glyceric acid. Oleic acid [*cis*-9-octadecenoic acid; 18:1(9c)] is the most common monounsaturated fatty acid in animals and vegetables. It is incorporated into the membranes of lactic acid bacteria grown in a medium, but is not synthesized (Johnsson et al., 1995). In *L. plantarum*, our metabolomic analysis indicated that the intracellular abundance of oleic acid decreased, while the extracellular abundance increased with RGDF supplementation. These findings suggest that oleic acid might be less incorporated from the medium, possibly by modified membrane rigidity by RGDF. Nicotinic acid, also known as niacin, is a form of vitamin B3 and is an essential human nutrient that can be supplied by plants and bacteria. Several cellular processes require the compound as a component of the coenzymes nicotinamide adenine dinucleotide (NAD) and NAD phosphate (NADP). In probiotic *Lactobacillaceae* spp., free nicotinic acid decrease with increasing cellular activity as it is largely incorporated in the form of cofactors (McIlwain et al., 1949). Nicotinic acid is also an important cofactor for lactate dehydrogenase, acting as the limiting factor for lactate production during fermentation, which might be associated with the reduced intracellular abundance and improved lactate production by RGDF (Colombié and Sablayrolles, 2004). Glyceric acid is a precursor of several phosphate derivatives that are important biochemical intermediates in glycolysis. 3-Phosphoglyceric acid is one derivative that is especially important for serine and cysteine biosynthesis. A recent study demonstrated that *L. plantarum* supplemented with 2% RGDF upregulates the expression of genes involved in serine (sdhA, sdhB, and sdaC) and cysteine metabolism (cysE) (Yu et al., 2022). Although further verification of the changes in specific metabolic and physiologic mechanisms is required, our results support the view that RGDF supplementation alters cellular and metabolic processes.

*Lactobacilli* are recognized for their ability to secrete many beneficial metabolites, such as SCFAs, indole-derivatives, and vitamins (Wang et al., 2018; Thompson et al., 2020; Sugimura et al., 2022). Our exometabolomic analysis revealed that 2-hydroxybutanoic acid, hexanol, and butyl acetate as metabolites that were secreted specifically in response to RGDF supplementation. These compounds are generally excreted as end products during propanoate biosynthesis and butanol metabolism. In mammalian tissues, 2-hydroxybutanoic acid, also known as α-hydroxybutyrate, is released as a byproduct when cystathionine is cleaved to cysteine for detoxification against oxidative stress. Although it has been used as a biomarker of type 2 diabetes and lactic acidosis, novel roles of 2-hydroxybutanoic acid have been suggested to protect against acetaminophen-induced liver injury and immune modulation against viral infection (Liu et al., 2018; Zheng et al., 2020; Shi et al., 2021). For example, the level of serum 2-hydroxybutanoic acid was reportedly enriched in patients with viral infections that included human papilloma virus or SARS-CoV-2 compared to healthy controls (Liu et al., 2018; Shi et al., 2021). It could be a result of the activation of antioxidant responses and control of cellular redox balance. Hexanol is an organic alcohol used in the perfume industry; its odor is that of freshly mown grass with a hint of strawberries. Its health-related functions are unclear, but it reportedly modulates the function of the actomyosin motor (Komatsu et al., 2004). Similar to hexanol, butyl acetate possesses characteristic flavors and a sweet odor of bananas or apples (Holland et al., 2005). It also has antimicrobial activity against undesirable microorganisms in cosmetic products, such as *Staphylococcus aureus* and *E. coli* (Lens et al., 2016). The specific mechanism of the secretion of these metabolites following stimulation by RGDF supplementation and comparative studies with FOS, would provide some evidence that metabolite production is highly specific to RGDF, but not to carbohydrate polymer-based dietary fiber. Further genetic investigations are required to elucidate the underlying mechanism.

## Conclusion

Red ginseng dietary fiber supplementation promoted probiotic properties of *L. plantarum*, including production of SCFAs (lactate and acetate), carbohydrate utilization, epithelial attachment, and pathogen inhibition. Comparative metabolomic analyses suggested RGDF-related modification of cellular and metabolic processes, including membrane biology and central carbon metabolism. In addition, the potential applications of bioactive compounds produced by RGDF-supplemented *L. plantarum* have been proposed as novel postbiotic metabolites.

## Data availability statement

The raw data supporting the conclusions of this article will be made available by the authors, without undue reservation.

## Author contributions

S-HY, YJ, and MS designed the study, and drafted and revised the manuscript. HJ, V-LT, J-HB, Y-JB, and RR performed the experiments, analyzed the data, and collected the samples and data interpretation. EN, S-KK, and W-SJ revised the manuscript and obtained the funding. All authors had read and approved the final manuscript.
