# Supplementary material for: Red ginseng dietary fiber promotes probiotic properties of Lactiplantibacillus plantarum and alters bacterial metabolism
Source: Front Microbiol. 2023 Mar 6;14:1139386. doi: 10.3389/fmicb.2023.1139386 (PMC10025373; doi:10.3389/fmicb.2023.1139386)

Supplementary Material

Red ginseng dietary fiber promotes probiotic properties of *Lactiplantibacillus plantarum* and alters bacterial metabolism

**Hyeon Ji Jeon^1, †^, Seung-Hwan You^2, †^, Eoun Ho Nam^3,4^, Van-Long Truong^1^, Ji-Hong Bang^1^, Yeon-Ji Bae^1^, Razanamanana. H. G. Rarison^1^, Sang-Kyu Kim^2^, Woo-Sik Jeong^1^, Young Hoon Jung^1, *^, Minhye Shin^3,4, *^**

*** Correspondence:** Young Hoon Jung, [younghoonjung@knu.ac.kr](mailto:younghoonjung@knu.ac.kr) and Minhye Shin, [mhshin@inha.ac.kr](mailto:mhshin@inha.ac.kr)

# Supplementary Figures and Tables

# Table S1. Lactate and acetate production of probiotic strains (*L. reuteri, L. plantarum, L. acidophilus, L. casei and L. lactis*)

# Table S2. List of metabolites identified in this study.

# Table S3. Growth and fermentation profiles of *L. platnarum* and *L. reuteri* when cultured in MRS broth, 0.5% RGDF and 0.5% FOS.

# Figure S1. Relative normalized abundance of intracellular metabolites of *L. plantarum* cultured with 0.5% (w/v) RGDF compared to the control MRS broth. Data are expressed as violin plots of six determinations. Differences between metabolite abundances were all significant at a significance level of 95% (*) and 99% (**), as determined by the Student's t-test.

**Table S1.** Lactate and acetate production of probiotic strains (*L. reuteri*, *L. plantarum*, *L. acidophilus*, *L. casei* and *L. lactis*)

|  | *L. reuteri* | *L. plantarum* | *L. acidophilus* | *L. casei* | *L. lactis* |
| --- | --- | --- | --- | --- | --- |
| Lactate (g/L) | 71.46% | 130.68% | 114.94% | 84.64% | 103.61% |
| Acetate (g/L) | 71.09% | 126.68% | 114.25% | 105.96% | 115.35% |

**Table S2.** List of metabolites identified in this study.

| Compound Name | CAS Number | Retention Time (min) | Retention Index | Kovats  Retention Index | Estimated  Retention  Index | AI-predicted  Retention Index |
| --- | --- | --- | --- | --- | --- | --- |
| 1,2,3,4,5,6-Hexa-o-trimethelsilyl-myo-inositol | [2582-79-8](https://www.chemsrc.com/en/baike/1366810.html) | 53.204 | 2020.7 | 2152 | 2194 | 1982 |
| 1,2,3,4,6-Penta-trimethylsilyl Glucopyranose | [19126-99-9](https://www.chemsrc.com/en/baike/9796.html) | 49.581 | 1960.3 | 1959 | 2037 | 1914 |
| 2,3-Dihydro-8-methoxyfuro(2,3-b)quinoline | 64124-80-7 | 53.881 | 2045.1 | - | 1769 | 1933 |
| 2,4-Dichloroaniline | [554-00-7](https://www.chemsrc.com/en/baike/402087.html) | 28.725 | 1493.8 | 1286 | 1352 | 1325 |
| 2-Deoxytetronic acid | 55191-53-2 | 27.844 | 1459.6 | 1453 | 1298 | 1438 |
| 2-Ethyl-1-butanol | 17888-61-8 | 17.54 | 1093.0 | 933 | 838 | 945 |
| 2-Hydroxybutanoic acid | 55133-93-2 | 23.797 | 1306.8 | 1136 | 1014 | 1148 |
| 2-Hydroxyhexanoic acid | 54890-07-2 | 22.558 | 1263.0 | 1284 | 1213 | 1292 |
| 2-Hydroxyisobutyric acid | 55133-92-1 | 19.368 | 1153.8 | 1071 | 994 | 1112 |
| 2-Hydroxypyridine | 142-08-5 | 16.872 | 1070.8 | 1094 | 847 | 1115 |
| 2-Trimethylsiloxy-1,2,3-propanetricarboxylic acid tris(trimethylsilyl) ester | [14330-97-3](https://www.chemsrc.com/en/baike/1369346.html)  (PubChem) | 42.668 | 1849.9 | 1839 | - | - |
| 3-Hydroxy-2,4,4-trimethylpentyl 2-methylpropanoate | [Not A](https://www.chemsrc.com/en/baike/1638477.html)vailable  (NIST#: 306325) | 14.362 | 987.4 | - | 1097 | 1110 |
| 3-Hydroxypropionic acid | 55162-32-8 | 20.015 | 1175.3 | 1142 | 979 | 1149 |
| 3-Methyl-2-pyrazoline | 108-26-9 | 15.128 | 1012.8 | - | 872 | 1099 |
| 4-Hydroxypyridine | [626-64-2](https://www.chemsrc.com/en/baike/1192397.html) | 20.027 | 1175.7 | 1154 | 895 | 1176 |
| 4-Methylamino-2(5h)-furanone | Not Available  (NIST#: 427455) | 14.549 | 993.6 | - | 1095 | 1371 |
| 5,6-Dihydrouracil | [504-07-4](https://www.chemsrc.com/en/baike/1105412.html) | 19.923 | 1172.2 | - | 1046 | 1598 |
| 5-Nonanone | [502-56-7](https://www.chemsrc.com/en/baike/401903.html) | 15.821 | 1035.9 | 1059 | 1052 | 1064 |
| Adenine | 73-24-5 | 45.406 | 1906.0 | 1836 | 1512 | 1797 |
| Adenosine | [58-61-7](https://www.chemsrc.com/en/baike/85738.html) | 64.65 | 2673.6 | - | 2589 | 2706 |
| Alanine | 56-41-7 | 18.738 | 1132.8 | - | 855 | 1239 |
| Aminomalonate | 959080-51-4 | 28.929 | 1501.5 | 1479 | 1413 | 1447 |
| Ascorbic acid | [50-81-7](https://www.chemsrc.com/en/baike/573142.html) | 41.363 | 1821.1 | 2120 | 1632 | 1721 |
| Asparagine | 55649-62-2 | 35.922 | 1703.6 | 1666 | 1745 | 1637 |
| Aspartic acid | [15985-05-4](https://www.chemsrc.com/en/baike/1581187.html) | 30.302 | 1547.7 | 1536 | 1512 | 1516 |
| Beta-alanine | [55255-77-1](https://www.chemsrc.com/en/baike/1195772.html) | 24.697 | 1340.1 | 1429 | 1161 | 1440 |
| Butyl phenyl ether | [1126-79-0](https://www.chemsrc.com/en/baike/754703.html) | 14.403 | 988.7 | 1169 | 1168 | 1195 |
| Chlorpheniramine | [25523-97-1](https://www.chemsrc.com/en/baike/233261.html) | 55.479 | 2103.1 | 2004 | 2023 | 1990 |
| Citric acid | [77-92-9](https://www.chemsrc.com/en/baike/1027486.html) | 42.678 | 1850.1 | 2261 | 1779 | 2110 |
| Creatinine | [60-27-5](https://www.chemsrc.com/en/baike/951433.html) | 31.61 | 1591.8 | - | 835 | 1745 |
| Cysteamine | [60-23-1](https://www.chemsrc.com/en/baike/584858.html) | 14.111 | 979.0 | - | 767 | 762 |
| D-Glucosamine | [943622-44-4](https://www.chemsrc.com/en/baike/1374792.html) | 46.938 | 1926.0 | 1866 | 1996 | 1888 |
| Diethanolamine | 20836-41-3 | 28.939 | 1501.8 | 1428 | 1244 | 1435 |
| Diisoamyl ether | [544-01-4](https://www.chemsrc.com/en/baike/1085295.html) | 14.194 | 981.8 | 1000 | 963 | 1023 |
| DL-alpha-Hydroxybutyric acid | [55133-93-2](https://www.chemsrc.com/en/baike/259600.html) | 19.371 | 1153.9 | 1130 | 1014 | 1148 |
| Dodecane | [112-40-3](https://www.chemsrc.com/en/baike/1034478.html) | 21.923 | 1240.8 | 1200 | 1214 | 1210 |
| Ethanolamine | 17165-52-5 | 14.116 | 979.2 | 1021 | 911 | 1045 |
| Fructose | 19126-98-8 | 45.285 | 1904.5 | 1863 | 1982 | 1895 |
| Fumaric acid | [110-17-8](https://www.chemsrc.com/en/baike/118568.html) | 25.858 | 1383.2 | - | 1140 | 1232 |
| GABA | [39508-23-1](https://www.chemsrc.com/en/baike/766256.html) | 30.759 | 1563.1 | 1542 | 1260 | 1536 |
| Gluconic acid | 526-95-4 | 50.177 | 1968.1 | - | 1866 | 1936 |
| Glucono-1,5-lactone | [90-80-2](https://www.chemsrc.com/en/baike/1474066.html) | 43.679 | 1872.2 | - | 1813 | 1681 |
| Glutamic acid | 617-65-2 | 33.719 | 1647.8 | - | 1410 | 2263 |
| Glyceric acid | 38191-87-6 | 25.167 | 1357.6 | 1343 | 1199 | 1343 |
| Glycerol | 6787-10-6 | 23.609 | 1299.8 | 1292 | 1108 | 1273 |
| Glycine | [56-40-6](https://www.chemsrc.com/en/baike/311698.html) | 24.698 | 1340.2 | - | 819 | 1454 |
| Glycolic acid | [79-14-1](https://www.chemsrc.com/en/baike/509829.html) | 17.907 | 1105.2 | 932 | 819 | 1025 |
| Heptacosane | [593-49-7](https://www.chemsrc.com/en/baike/951301.html) | 64.272 | 2651.5 | 2700 | 2705 | 2731 |
| Hexadecane | [544-76-3](https://www.chemsrc.com/en/baike/171948.html) | 30.1 | 1540.9 | 1600 | 1612 | 1610 |
| Hexanol | 111-27-3 | 14.032 | 976.4 | 854 | 860 | 867 |
| Hexyl pivalate | [5434-57-1](https://www.chemsrc.com/en/baike/171708.html) | 15.712 | 1032.2 | 1163 | 1197 | 1161 |
| Homoserine | [498-19-1](https://www.chemsrc.com/en/baike/78171.html) | 28.331 | 1478.5 | - | 1197 | 1962 |
| Hydroxylamine | [21023-20-1](https://www.chemsrc.com/en/baike/121857.html) | 19.179 | 1147.5 | 1111 | 771 | 1165 |
| Isoleucine | [73-32-5](https://www.chemsrc.com/en/baike/1614184.html) | 24.287 | 1324.9 | - | 1089 | 1474 |
| Lactic acid | 17596-96-2 | 17.369 | 1087.3 | 1061 | 915 | 1077 |
| Leucine | [61-90-5](https://www.chemsrc.com/en/baike/757034.html) | 24.342 | 1327.0 | - | 1089 | 1535 |
| L-Malic acid | 6915-15-7 | 29.345 | 1515.5 | - | 1294 | 1592 |
| L-Norleucine | [327-57-1](https://www.chemsrc.com/en/baike/958114.html) | 23.679 | 1302.4 | - | 1153 | 1591 |
| L-Norvaline | [760-78-1](https://www.chemsrc.com/en/baike/253848.html) | 14.383 | 988.1 | - | 1054 | 1524 |
| L-Pyroglutamic acid | [98-79-3](https://www.chemsrc.com/en/baike/123373.html) | 30.663 | 1559.9 | 1671 | 1180 | 1662 |
| Lysine | 56-87-1 | 44.465 | 1889.5 | - | 1396 | 1858 |
| Malonic acid | [141-82-2](https://www.chemsrc.com/en/baike/1028701.html) | 19.609 | 1161.8 | - | 1032 | 1401 |
| Mannose | 6736-99-8 | 46.002 | 1913.8 | 1844 | 1970 | 1893 |
| Methionine | [27844-10-6](https://www.chemsrc.com/en/baike/343553.html) | 30.48 | 1553.7 | 1528 | 1487 | 1536 |
| Methyldopa | [555-30-6](https://www.chemsrc.com/en/baike/895359.html) | 57.853 | 2204.6 | - | 2048 | 2209 |
| Nicotinic acid | [59-67-6](https://www.chemsrc.com/en/baike/1027482.html) | 24.653 | 1338.5 | 1242 | 1144 | 1230 |
| Nonadecane | [629-92-5](https://www.chemsrc.com/en/baike/173580.html) | 36.628 | 1718.8 | 1900 | 1910 | 1918 |
| Nonanol | [143-08-8](https://www.chemsrc.com/en/baike/750869.html) | 21.677 | 1232.2 | 1157 | 1159 | 1171 |
| Octadecanol acetate | [822-23-1](https://www.chemsrc.com/en/baike/996817.html) | 54.59 | 2070.6 | 2192 | 2177 | 2228 |
| Octamethylcyclotetrasiloxane | [556-67-2](https://www.chemsrc.com/en/baike/442659.html) | 14.84 | 1003.3 | 1010 | 827 | 990 |
| Octanol acetate | [112-14-1](https://www.chemsrc.com/en/baike/829797.html)  (PubChem) | 16.465 | 1057.3 | 1185 | - | - |
| Oleic acid | [112-80-1](https://www.chemsrc.com/en/baike/895631.html) | 56.879 | 2162.6 | 2116 | 2175 | 2172 |
| Ornithine | 24595-70-8 | 33.545 | 1643.3 | 1767 | 1643 | 1620 |
| Palmitic acid | [57-10-03](https://www.chemsrc.com/en/baike/80973.html) | 52.253 | 1995.1 | 1954 | 1968 | 1993 |
| Phenylalanine | 63-91-2 | 31.74 | 1596.2 | 1625 | 1528 | 1695 |
| Picolinic acid | [98-98-6](https://www.chemsrc.com/en/baike/26107.html) | 14.064 | 977.5 | - | 1144 | 1229 |
| Pinitol | [484-68-4](https://www.chemsrc.com/en/baike/1112311.html)  (PubChem) | 42.981 | 1856.8 | 1880 | - | - |
| P-menthan-4-ol | [470-65-5](https://www.chemsrc.com/en/baike/1215987.html) | 18.687 | 1131.2 | 1135 | 1127 | 1183 |
| Proline | 7364-47-8 | 24.541 | 1334.4 | 1305 | 1258 | 1314 |
| Propyleneglycol | 17887-27-3 | 15.496 | 1025.1 | 1006 | 824 | 1000 |
| Pyrene | [129-00-0](https://www.chemsrc.com/en/baike/338143.html) | 53.875 | 2044.8 | 2068 | 1984 | 2128 |
| Pyridine | [110-86-1](https://www.chemsrc.com/en/baike/752647.html) | 14.061 | 977.4 | 1186 | 674 | 730 |
| Raffinose | [34141-00-9](https://www.chemsrc.com/en/baike/904526.html) | 75.201 | 3419.4 | - | 3879 | 3281 |
| Ribitol | [32381-53-6](https://www.chemsrc.com/en/baike/519609.html) | 43.661 | 1871.8 | 1727 | 1746 | 1771 |
| Ribose | [33648-69-0](https://www.chemsrc.com/en/baike/684565.html) | 44.843 | 1897.8 | 1648 | 1651 | 1660 |
| Sec-butyl acetate | [105-46-4](https://www.chemsrc.com/en/baike/829786.html) | 16.472 | 1057.5 | 987 | 721 | 757 |
| Serine | 64625-17-8 | 23.341 | 1290.4 | 1355 | 1322 | - |
| Shikimic acid | [55520-78-0](https://www.chemsrc.com/en/baike/894790.html) | 52.741 | 2004.0 | 1843 | 1904 | 1847 |
| Sorbitol | 14199-80-5 | 47.35 | 1931.3 | 1974 | 2066 | - |
| Sorbose | 30645-02-4 | 45.281 | 1904.4 | - | 2049 | 1835 |
| Stearic acid | [57-11-04](https://www.chemsrc.com/en/baike/951972.html) | 57.483 | 2188.3 | 2172 | 2167 | 2197 |
| Succinic acid | [110-15-6](https://www.chemsrc.com/en/baike/401712.html) | 24.9 | 1347.7 | - | 1132 | 1301 |
| Sulfuric acid | [7664-93-9](https://www.chemsrc.com/en/baike/668855.html) | 20.596 | 1194.6 | 1148 | 1096 | 1200 |
| Tetradecane | [629-59-4](https://www.chemsrc.com/en/baike/898084.html) | 27.404 | 1442.5 | 1400 | 1413 | 1408 |
| Threonine | [64659-35-3](https://www.chemsrc.com/en/baike/895099.html) | 24.37 | 1328.0 | 1396 | 1357 | 1390 |
| Tridecane | [629-50-5](https://www.chemsrc.com/en/baike/1191735.html) | 18.918 | 1138.8 | 1300 | 1313 | 1309 |
| Tridecanol | [112-70-9](https://www.chemsrc.com/en/baike/1117942.html) | 25.235 | 1360.1 | 1565 | 1556 | 1580 |
| Tryptophan | [73-22-3](https://www.chemsrc.com/en/baike/1035237.html) | 56.764 | 2157.7 | - | 1998 | 2349 |
| Tyramine | [68595-84-6](https://www.chemsrc.com/en/baike/509756.html) | 47.193 | 1929.3 | 1922 | 1657 | 1886 |
| Tyrosine | [7536-83-6](https://www.chemsrc.com/en/baike/766187.html) | 48.184 | 1942.2 | 1907 | 1845 | 1886 |
| Undecane | [1120-21-4](https://www.chemsrc.com/en/baike/830639.html) | 16.139 | 1046.4 | 1100 | 1115 | 1111 |
| Undecanol | [4272-06-4](https://www.chemsrc.com/en/baike/1658977.html) | 18.351 | 1120.0 | - | 1277 | 1287 |
| Uracil | 10457-14-4 | 25.581 | 1372.9 | 1345 | 1264 | 1327 |
| Urea | [60615-84-1](https://www.chemsrc.com/en/baike/1042323.html) | 14.331 | 986.3 | 1194 | 1195 | 1182 |
| Valine | [15984-93-7](https://www.chemsrc.com/en/baike/524748.html) | 22.103 | 1247.1 | - | 1172 | 1222 |
| Xylonic acid | [Not A](https://www.chemsrc.com/en/baike/399450.html)vailable  (NIST Chemistry WebBook) | 40.678 | 1806.0 | 1818 | - | - |

**Table S3.** Growth and fermentation profiles of *L. platnarum* and *L. reuteri* when cultured in MRS broth, 0.5% RGDF and 0.5% FOS.


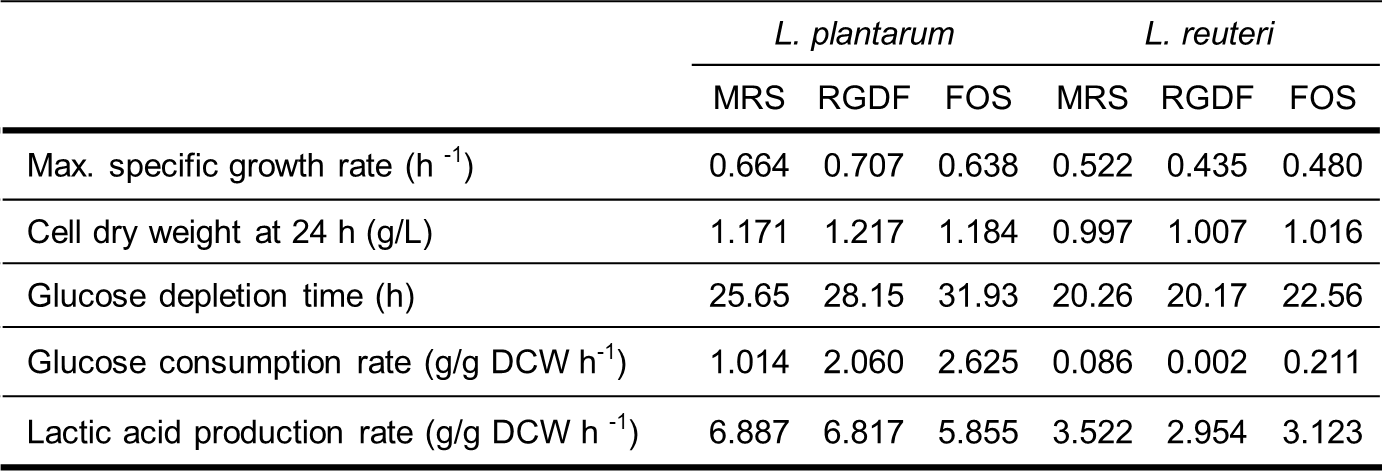


**Figure S1.** Relative normalized abundance of intracellular metabolites of *L. plantarum* cultured with 0.5% (w/v) RGDF compared to the control MRS broth. Data are expressed as violin plots of six determinations. Differences between metabolite abundances were all significant at a significance level of 95% (*) and 99% (**), as determined by the Student's t-test.


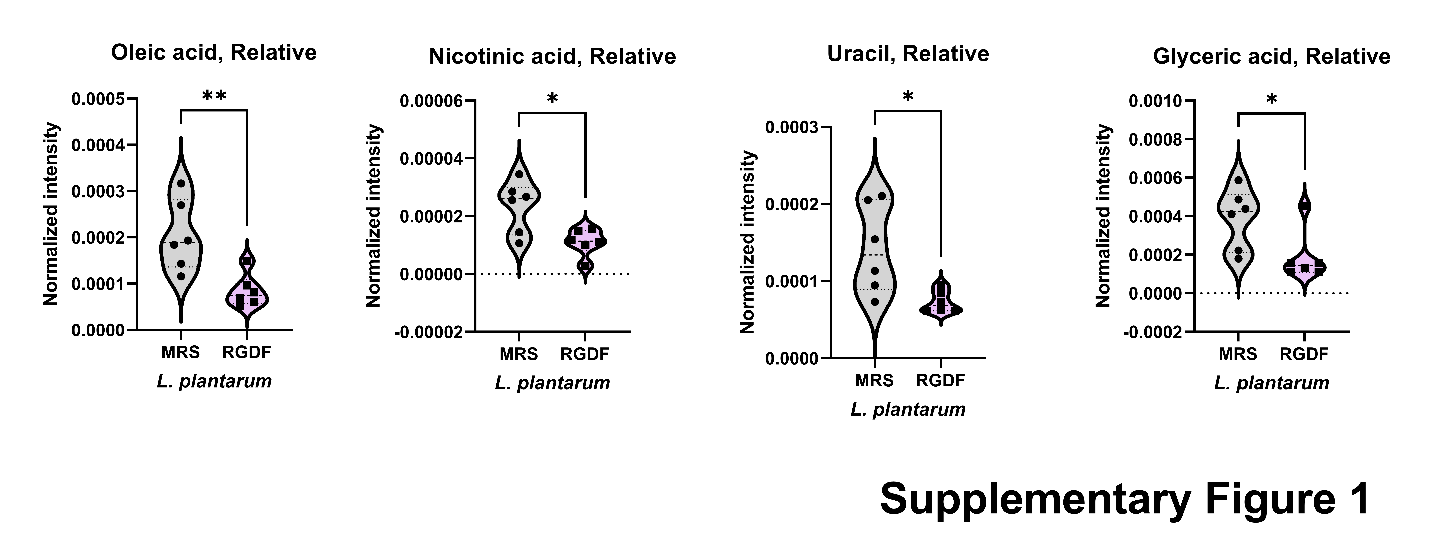

Supplement: Supplementary file 1 [file Data_Sheet_1.docx]
